# One-stage reconstruction using a fibula osteocutaneous free flap and an anterolateral thigh free flap for an extensive composite defect after en bloc resection of squamous cell carcinoma on the mouth floor, mandible, and anterior neck: A CARE-compliant case report

**DOI:** 10.1097/MD.0000000000033786

**Published:** 2023-05-26

**Authors:** SeHo Shin, KiHyun Kim, SangSeok Woo, KyungMin Kim, JunWon Lee, SeongHwan Kim, JaiKoo Choi, DongJin Lee, InSuck Suh

**Affiliations:** a Department of Plastic and Reconstructive Surgery, Kangnam Sacred Heart Hospital, College of Medicine, Hallym University, Seoul, Korea; b Department of Otorhinolaryngology, Kangnam Sacred Heart Hospital, College of Medicine, Hallym University, Seoul, Korea.

**Keywords:** mandibular reconstruction, mouth floors, three-dimensional printings

## Abstract

Although regional metastasis to the lymph nodes is common in advanced oral cancer, extensive local invasion into surrounding structures such as the mandible, skin and soft tissue of the neck, and masticator space is relatively rare. Sometimes surgical treatment cannot be performed and only palliative chemotherapy and radiation therapy are offered to preserve the quality of life of patients with advanced oral cancer. Nevertheless, the surgical resection of tumors remains the most effective treatment. This study presents a case of aggressive mouth floor cancer in which extensive composite defects on the mouth floor, oral mucosa, mandible, skin and soft tissue of the neck caused by tumor resection were reconstructed.

**Patient concerns:**

A 66-year-old man and a 65-year-old man with no significant personal or family history visited our clinic due to a large and multiple masses on the floor of the mouth and both sides of the neck.

**Diagnosis:**

Histopathological evaluation of the biopsy specimen revealed squamous cell carcinoma.

**Interventions:**

A fibula osteocutaneous free flap and customized titanium plate were used for the intraoral lining. Mandibular reconstruction was performed using a 3D-printed bone model, and an anterolateral thigh free flap was used to resurface the anterior of the neck.

**Outcomes:**

Reconstruction using this method was successful, and excellent functional and aesthetic outcomes were achieved without cancer recurrence.

**Lessons:**

This study show that the reconstruction of extensive composite defects of the oral mucosa, mandible, and neck soft tissue following surgical resection of mouth floor cancer can be performed in a single-stage operation. Through a single-stage reconstruction, both excellent functional aspects without cancer recurrence and satisfactory aesthetic outcomes can be obtained.

## 1. Introduction

Mouth floor cancer is an oral cavity cancer. In South Korea, the incidence of head and neck cancers (HNCs), including mouth floor cancer, is steadily increasing, although a fluctuating trend has been observed worldwide (i.e., an increasing-stagnating-decreasing-increasing-stagnating incidence pattern).^[1,2]^ Squamous cell carcinomas (SCC) account for > 90% of oral cancer cases, mostly located on the tongue and mouth floor.

Although regional metastasis to the lymph nodes is common in advanced oral cancer, extensive invasion into surrounding structures such as the mandible, skin and soft tissue of the neck, and the masticator space is relatively rare.^[2–4]^ In cases of advanced oral cancer, the characteristics of the cancer defects and the patient’s general condition sometimes prohibit surgical resection, and palliative chemotherapy and radiation therapy are the only options to preserve quality of life.

Nonetheless, the most effective treatment for complete remission is surgical resection of the tumor.^[4–6]^ The extensive composite defects in the soft tissues of the oral cavity, neck, and mandible left by tumor resection are not easy to reconstruct, and more than one free vascularized tissue transfer is sometimes required for reconstruction.^[7–9]^ To reconstruct composite defects, various free flap techniques have been used with grafts harvested from a variety of donor sites.^[10]^

This study presents 2 cases of mouth floor cancer for which one-stage reconstruction of extensive composite defects of the mouth floor, oral mucosa, mandible, and skin and soft tissue of the neck was undertaken, following en bloc resection and neck dissection. Fibula osteocutaneous free flaps and customized titanium plates were used for the intraoral lining and mandibular reconstruction. Three-dimensional (3D)-printed bone models were used to guide mandibular reconstruction. The anterior neck was resurfaced using an anterolateral thigh (ALT) free flap. Both one-stage reconstructions using a fibula osteocutaneous free flap and an ALT free flap were successful, without complications such as hematoma, plate exposure, infection, or problems with speaking and swallowing.

## 2. Case 1

**Figure 1.**
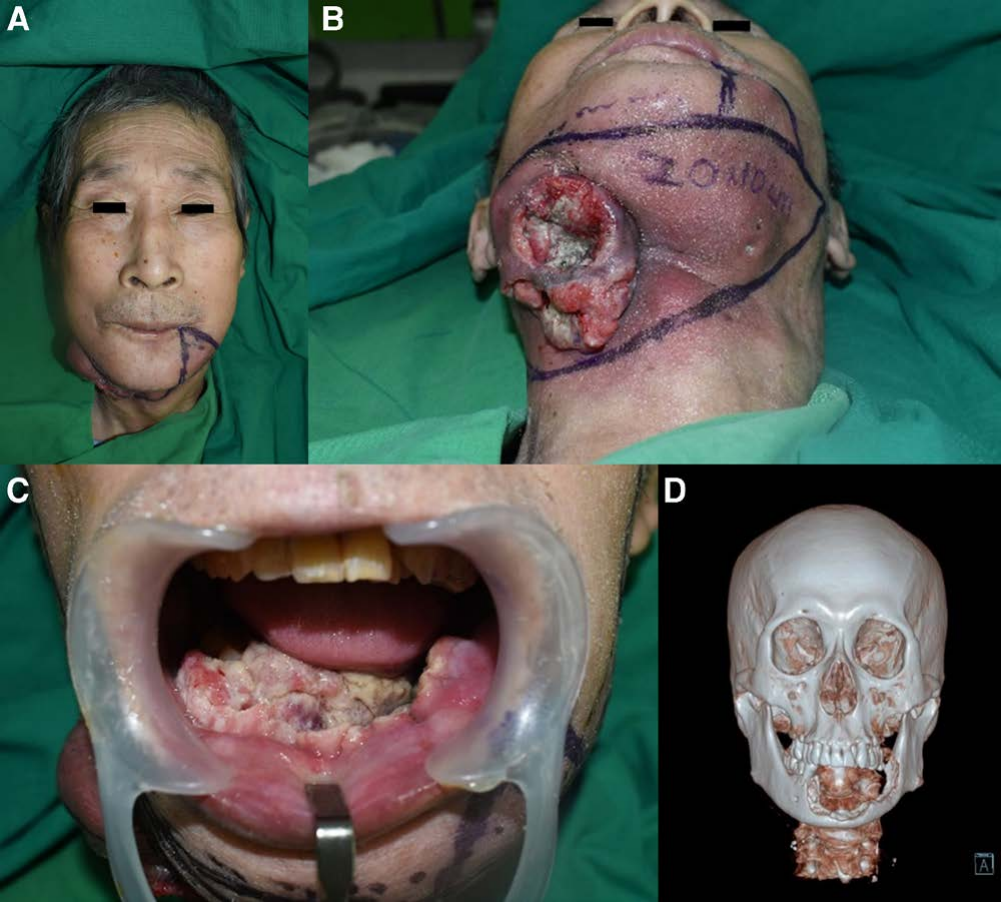
Preoperative clinical photograph and computed tomography scan. (A) Front view. (B) The primary mass at the floor of the mouth. (C) The primary mass extended to the tongue, oral mucosa, and mandibular bone. (D) Mandibular bone destruction by invading squamous cell carcinoma.

A 66-year-old man was seen in our clinic for a large mass on the floor of the mouth that had developed 6 months prior to being seen at the Department of Otorhinolaryngology. The mass had been identified as an SCC after incisional biopsy. The primary mass was in the anterior aspect of the floor of the mouth and was approximately 7 cm × 4 cm. It was accompanied by bone destruction in the mandibular parasymphysis, symphysis, and body according to preoperative computed tomography (CT) and magnetic resonance imaging. In addition, the mass had metastasized to the cervical lymph nodes, where masses approximately 4 cm × 3 cm and 3 cm × 3 cm were found at neck levels Ib and III, respectively. There was no distant metastasis. The primary tumor extended into the skin and soft tissue of the right submandibular area and formed an elevated, hard palpable mass of approximately 9 cm × 8 cm that had ulcerations with irregular margins, a foul odor, and greenish discharge (Fig. 1).

After en bloc resection of the oral SCC, reconstruction of the mandible and mouth floor was planned, using a fibula osteocutaneous free flap with a customized titanium plate. Neck resurfacing was also planned, using an anterolateral thigh free flap.

A 3D bone model was designed using preoperative CT images to accurately determine the extent of resection and the amount of bone to harvest. Images of the mandibular bone were converted to 3D models using Mimics software (Materialise, Leuven, Belgium), and a bone model was manufactured using VisiJet® PXL core (calcium sulfate hemihydrate; 3D Systems, Rock Hill, SC) and VisiJet® PXL clear (2-pyrrolidone; 3D Systems) printing materials.

A partial glossectomy, a segmental mandibulectomy, and a modified radical neck dissection (MRND) were undertaken by otorhinolaryngologic surgeons for en bloc resection of the primary tumors that extended to the soft tissue of the neck and the metastasized lymph nodes.

During en bloc resection of the oral SCC, we simultaneously raised a right fibula free flap in a two-team procedure. The lateral approach first described by Gilbert was used.^[11]^ The 3D-printed bone model allowed the surgeons to precisely determine where to perform the mandibulectomy, using both mandibular angles. Thus, it was possible to predict the size of the defect that would be created by the mandibulectomy. Given the size of the defect, a fibula free flap based on a peroneal vessel was raised with a 19 cm × 7 cm skin paddle along the 16 cm section of fibular bone, including 1 septocutaneous perforator. The customized titanium plate was also pre-molded according to the bone model, and the fibular bone was divided into 3 sections measuring 6 cm, 4 cm, and 6 cm. After vertical mandibulectomy at both mandibular angles, the remaining ramus and plate were fixed with a titanium screw, and the osseous segments of the fibula were also fixed. The flap was properly affixed to the right mandibular defect since it had been molded prior to surgery. The skin paddle was reconstructed for the intraoral lining. Microvascular anastomosis was then performed using end-to-end anastomosis of the peroneal artery to the left facial artery and of the venae comitantes to branches of the left external jugular vein. In this way, the direction of the donor site and the recipient site was the same, and the skin paddle was used to reconstruct the intraoral lining (RRI group: Right side of defect, Right side of defect, skin paddle of fibula flap for Intraoral lining). The pedicle formed a gentle curvature to prevent vascular kinking.^[12]^

**Figure 2.**
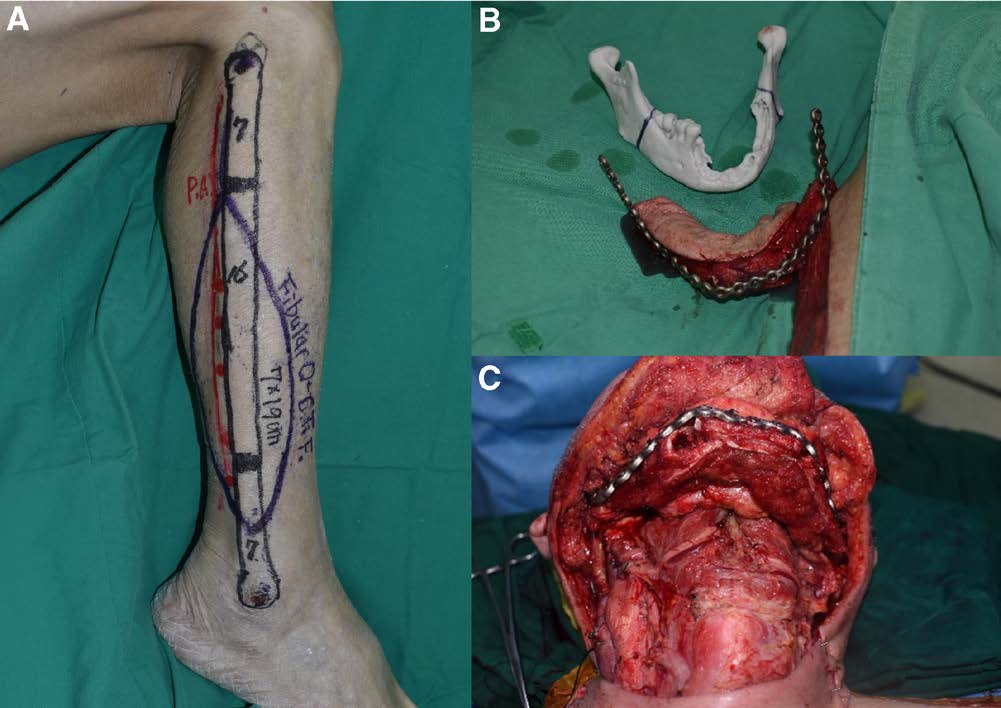
Intraoperative clinical photograph. The extensive mandibular bone defect was reconstructed using a segmented fibula free flap with a customized titanium plate based on a 3D-printed bone model. (A) Preoperative design of fibula osteocutaneous free flap. (B) Segmented fibula free flap with a plate and a 3D-printed bone model. (C) Internal fixation with segmented flap and plate.

During the anastomosis process mentioned above, the ALT free flap was also harvested. The skin paddle was 10 cm × 23 cm, designed to correspond to the expected defect size created by the MRND. Two musculocutaneous perforators were included in the skin paddle, and dissection reached the descending branch of the lateral circumflex femoral artery to ensure a pedicle length of approximately 7 cm. The defect and the flap were approximately the same size. Microvascular anastomosis was performed using end-to-end anastomosis of the descending branch of the lateral circumflex femoral artery to the right superior thyroid artery and of the venae comitantes to branches of the internal jugular vein (Fig. 2).

**Figure 3.**
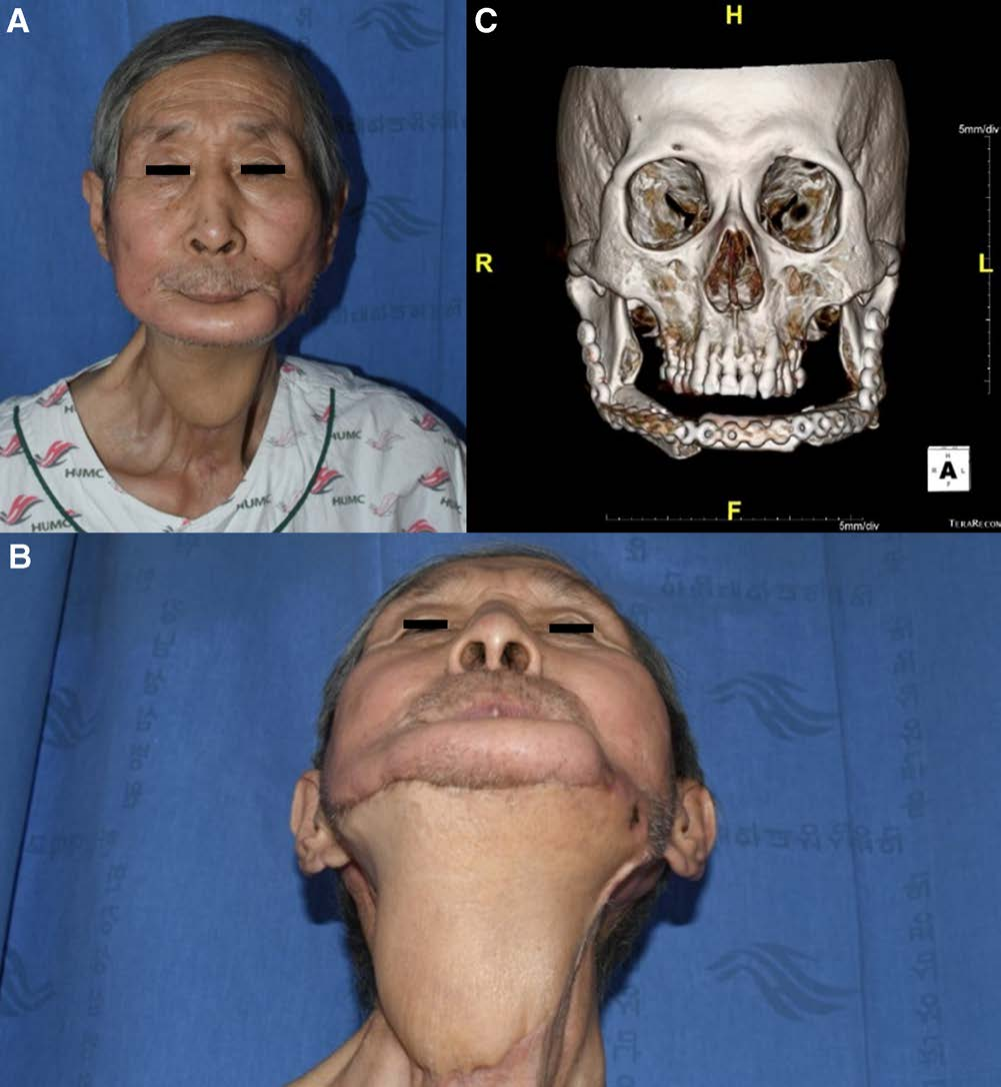
Patient at 20 months postoperatively. Excellent functional outcomes with good speech and swallowing and satisfactory aesthetic outcomes were achieved without flap bulkiness, scar contracture, or cancer recurrence. (A) Front view. (B) Worm’s eye view. (C) Computed tomography. The customized titanium plate was well fixed with osseous segments of the fibula free flap.

Early complications such as flap necrosis, hematoma, and seroma were not observed. At 20 months postoperatively, excellent functional outcomes, including speech and swallowing, and satisfactory aesthetic outcomes were achieved without flap bulkiness, scar contracture, or cancer recurrence (Fig. 3).

## 3. Case 2

**Figure 4.**
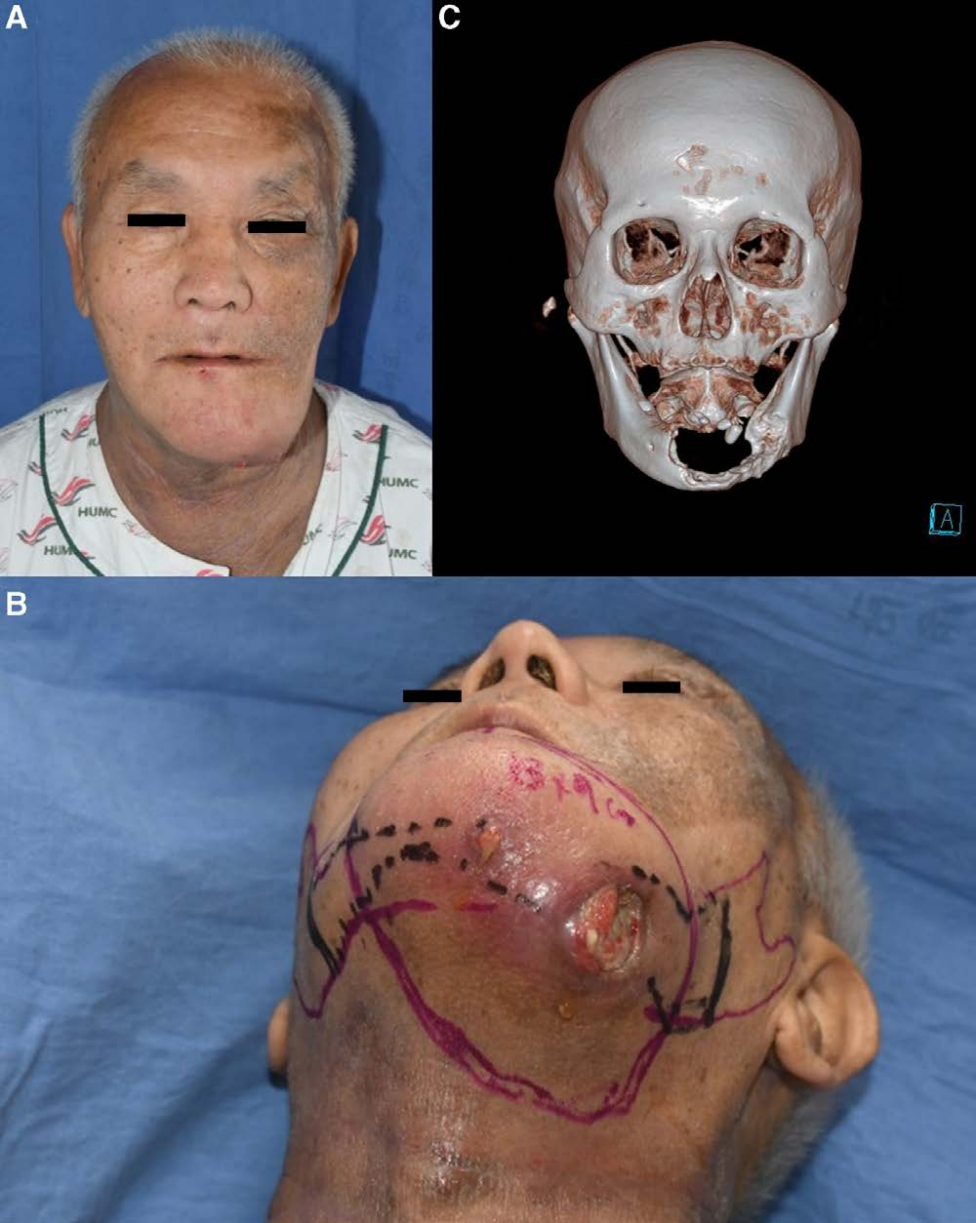
Preoperative clinical photograph and computed tomography scan. (A) Front view. (B) The ulcerative mass on the left neck extended to the tongue, oral mucosa, and mandibular bone. (C) Mandibular bone destruction by invading squamous cell carcinoma.

A 65-year-old man was seen in our clinic for an ulcerative lesion on his left chin that had developed 2 months prior to a consultation at the Department of Otorhinolaryngology. The mass had been identified as SCC after an incisional biopsy. The primary mass was in the anterior aspect of the mouth floor and was approximately 9 cm × 5 cm. The mass was accompanied by bone destruction in the mandibular parasymphysis, symphysis, and body according to preoperative CT and magnetic resonance imaging. There was no regional or distant metastasis. The primary tumor extended into the skin and soft tissue of the left submandibular area and formed an elevated, hard palpable mass of approximately 7 cm × 5 cm with ulcerations that had irregular margins, a foul odor, and greenish discharge (Fig. 4).

We reconstructed the defect in the same way as in the previous case. A fibula osteocutaneous free flap and an ALT free flap in the same direction as the expected defect were planned, and a 3D-printed bone model was used. A segmental mandibulectomy, and a selective neck dissection (SND; right: levels I-III, left: levels I-IV) were undertaken by otorhinolaryngologic surgeons for en bloc resection of the primary tumor that extended into the soft tissue of the neck.

Given the size of the defect, a fibula free flap based on a peroneal vessel was raised with a 13 cm × 8 cm skin paddle along the 16-cm fibular bone that included 2 septocutaneous perforators.

The customized titanium plate was pre-molded according to the bone model, and the fibular bone was divided into 3 sections measuring 6 cm, 4 cm, and 6 cm. After vertical mandibulectomy at both mandibular angles, the remaining ramus and plate were fixed with a titanium screw, and the osseous segments of the fibula were also fixed. The flap was properly affixed to the left mandibular defect since it was molded prior to surgery. Microvascular anastomosis was then performed using end-to-end anastomosis of the peroneal artery to the right superior thyroid artery and of the venae comitantes to branches of the left external jugular vein. The directions of the donor site and the recipient site were the same. The skin paddle was reconstructed for the intraoral lining (LLI group: Left side of defect, Left side of defect, skin paddle of fibula flap for Intraoral lining), and the pedicle formed a gentle curvature.^[12]^

**Figure 5.**
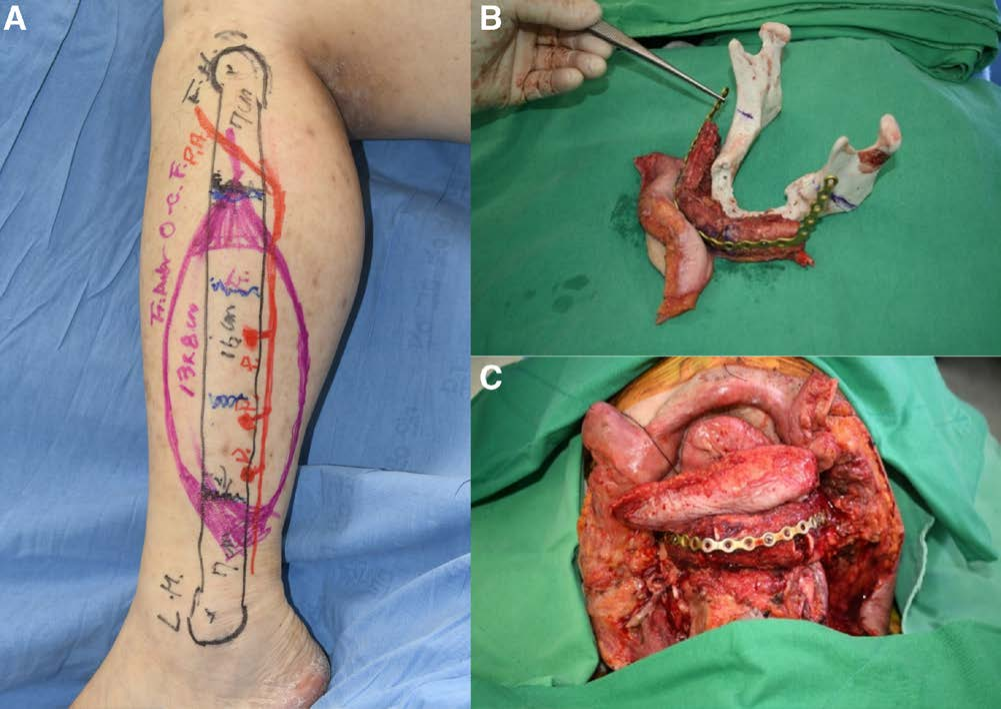
Intraoperative clinical photograph. The extensive mandibular bone defect was reconstructed using a segmented fibula free flap with a customized titanium plate based on a 3D-printed bone model. (A) Preoperative design of fibula osteocutaneous free flap. (B) Segmented fibula free flap with a plate and a 3D-printed bone model. (C) Internal fixation with segmented flap and plate.

During the anastomosis process, the planned ALT free flap was harvested. The skin paddle was designed to be 14 cm × 8 cm to correspond to the expected defect size after en bloc resection of the tumor and SND. Two musculocutaneous perforators were included in the skin paddle. The size of the defect and the flap were approximately the same, and microvascular anastomosis was performed using end-to-end anastomosis of the descending branch of the lateral circumflex femoral artery to the left superior thyroid artery and of the venae comitantes to the branches of the left external and internal jugular veins (Fig. 5).

**Figure 6.**
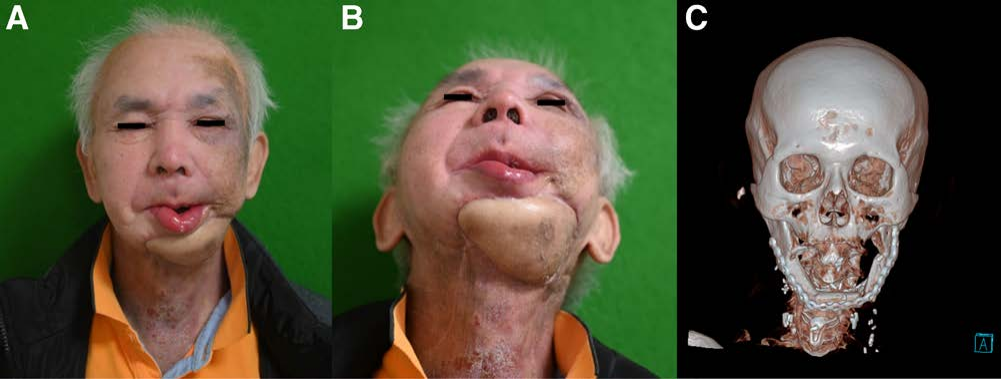
Patient at 6 months postoperatively. Excellent functional outcomes with good speech and swallowing and satisfactory aesthetic outcomes were achieved without flap bulkiness, scar contracture, or cancer recurrence. (A) Front view. (B) Worm’s eye view. (C) Computed tomography. The customized titanium plate was well fixed with osseous segments of the fibula free flap.

At 6 months postoperatively, excellent functional outcomes, including speech and swallowing, and satisfactory aesthetic outcomes were achieved without flap bulkiness, scar contracture, or cancer recurrence (Fig. 6).

## 4. Discussion

Mouth floor cancer is one of several oral cavity cancers within the category of HNCs. Although the incidence of HNCs varies from country to country, it is the sixth most common cancer worldwide.^[2,13,14]^ The incidence of all HNCs except laryngeal cancer is on the rise in South Korea. Although the incidence of HNCs among men is still much higher than it is among women, the incidence of HNCs in women is also increasing rapidly.^[1]^ In the past, alcohol consumption and tobacco use were seen as the major risk factors for HNCs, but human papillomavirus infection has recently been found to be a cause as well.^[1,14]^

SCC accounts for > 90% of oral cancer cases. When oral cancer is diagnosed at an early stage, the survival rate is 80% to 90%. However, if the initial diagnosis is made at an advanced stage, the survival rate may decrease, and the patient may require any combination of surgical treatment, chemotherapy, and radiation therapy. Although regional metastasis to the lymph nodes is common for advanced oral cancer, extensive invasion into surrounding structures such as the mandible and the skin and soft tissue of the neck and masticator space is relatively rare.^[2–4,15]^ Arya et al^[16]^ reported that the probability of mandibular erosion in advanced oral cancer was less than 10% and that masticator space spread was even rarer. Of the treatment options for advanced oral cancer, surgical excision is the most effective. However, if the disadvantages of surgical excision outweigh the advantages, palliative chemotherapy and radiation therapy are the only options for preserving the patient’s quality of life.^[5,6]^ The degree of treatment needed for advanced oral cancer varies depending on the extent of local invasion and regional metastasis. Following extensive resection of the primary tumor, additional mandibular resection and radical neck dissection may also be needed.^[15]^ Extensive composite defects may occur in the intraoral cavity, oral mucosa, jawbones, and soft tissues after en bloc resection of the tumor. Depending on the patient’s general condition and prognosis, surgery itself can be a substantial burden to the patient. Nonetheless, the main form of treatment for oral cancer remains surgical resection of the tumor.^[5,6,17]^

The goal of surgery is to completely remove cancerous tissue. Incomplete removal increases the risk of recurrence and lowers long-term survival. However, since excessive resection margins in oral cancer surgery can cause functional and aesthetic morbidities, clean resection margins of approximately 1 cm in three dimensions are considered appropriate.^[6]^

The reconstruction of composite defects after tumor resection is challenging. Specifically, mandibular bone defects caused by the removal of malignant tumors are usually accompanied by defects in the oral mucosa, which are the most problematic.^[7]^ While the overall purpose of oral reconstruction is like that of reconstruction in other areas, the main purpose is to restore normal anatomical structures and functions such as speaking and swallowing. Mandibular reconstruction has historically used methods such as bone grafts and bone marrow grafts. Currently, free vascularized tissue transfer is the most widely used procedure. Various donor sites can be used to create a free flap, but in recent years the most common donor sites have been the scapula, iliac crest, radial forearm, and fibula. No single donor site has an absolute advantage over the others, and the most suitable donor site should be selected based on factors such as the patient’s defect size and donor site morbidity.^[18]^ A fibula free flap based on the peroneal vessels is the most common method for mandibular reconstruction. The pedicle of the flap is sufficient in length and diameter and can be harvested from bones up to 30 cm long, making them suitable for reconstructing large defects.^[8,10,18]^ In addition, the peroneal vessel is located parallel to the fibular bone and provides a rich periosteal blood supply. This makes multiple-segment osteotomy possible, which is helpful for the reconstruction of various types of mandibular defects.^[10]^ Although peripheral vascular disease and ankle instability can lead to donor site morbidity in fibula free flaps, the incidence of these complications is low.^[7,18]^ Therefore, we selected a fibula free flap to reconstruct the mandibular defects in this study due to the size and location of the defects, the advantages of this flap type, and the low risk of donor-site morbidity.

A custom 3D-printed bone model was used to guide surgery. Advances in 3D printing technology enable medical personnel to create accurate physical 3D models based on patients’ imaging information.^[19]^ Reconstruction using 3D-printed models enables surgeons to preoperatively conduct surgery simulations based on the patient’s particular features, helping them better understand the patient’s condition and reduce the general anesthesia time. However, days or weeks may be needed to manufacture a 3D model, which requires collaboration between radiologists, engineers, and surgeons to create an accurate bone model.^[19,20]^ By creating a 3D-printed bone model, a prefabricated titanium plate can be molded and prepared to perfectly match the expected size of the patient’s defect.

After oral cancer surgery, patients often lose their teeth. Loss of teeth is inevitably accompanied by deterioration of function, such as maximum bite force and masticator performance, with a related diminishment of quality of life. Therefore, dental rehabilitation plans using conventional prostheses or implants should be established at the time of diagnosis. Although dental rehabilitation with implants is more effective, patients often decline it for economic reasons. Unfortunately, the patients in this study did not receive dental rehabilitation with implants for financial reasons.^[21]^

In addition to mandibular reconstruction after tumor removal and bilateral MRND, reconstruction of skin and soft tissue defects in the anterior neck was required. Due to the size, thickness, color, and texture of the defects, ALT fasciocutaneous free flaps were harvested for the immediate and simultaneous reconstruction of the composite defects. ALT free flaps have been widely used to rebuild composite defects that include soft tissue, mucosa, and bones in head and neck reconstruction. The most significant advantage of ALT free flaps is the ability to obtain a reliable skin paddle and a pedicle that is sufficient in length and diameter. In addition, anatomical variation in the pedicle is rare. Other advantages of this flap are the low risk of serious donor site morbidity and the ability to perform two-team operations.^[22]^

Neither patient had acute complications such as hematoma, seroma, or wound infection. Both had excellent functional outcomes related to speaking and swallowing, in addition to satisfactory aesthetic outcomes. Reconstruction in both cases was achieved without flap bulkiness, scar contracture, or cancer recurrence.

## 5. Conclusion

The purpose of this study was to demonstrate that the reconstruction of extensive composite defects in the oral mucosa, mandible, and neck soft tissue following surgical resection of mouth floor cancer can be performed in a single-stage operation. This single-stage reconstruction can achieve both excellent functional results, without cancer recurrence, and satisfactory aesthetic outcomes.

## Author contributions

**Conceptualization:** SeHo Shin, InSuck Suh, SeongHwan Kim, JaiKoo Choi, DongJin Lee.

**Data curation:** SeHo Shin, KiHyun Kim, SangSeok Woo, KyungMin Kim, JunWon Lee.

**Formal analysis:** SeHo Shin, KiHyun Kim, SangSeok Woo, KyungMin Kim, JunWon Lee, SeongHwan Kim, JaiKoo Choi, DongJin Lee.

**Project administration:** InSuck Suh.

**Supervision:** InSuck Suh, SeongHwan Kim, JaiKoo Choi, DongJin Lee.

**Writing – original draft:** SeHo Shin, InSuck Suh.

**Writing – review & editing:** SeHo Shin, InSuck Suh, SeongHwan Kim, JaiKoo Choi, DongJin Lee.
